# Botanical Origin-Dependent Phytochemical Profiles and Pharmaceutical Properties of Medical-Grade Honeys: Transdermal Delivery and Antibacterial Efficacy in a Wound Fluid Model

**DOI:** 10.3390/molecules31111863

**Published:** 2026-05-29

**Authors:** Anna Nowak, Wojciech Żwierełło, Izabela Gutowska, Anna Muzykiewicz-Szymańska, Edyta Kucharska, Jana Godocikova, Viktoriia Chirkova, Łukasz Kucharski, Katarzyna Piotrowska, Karolina Jakubczyk, Kinga Szymczykowska, Juraj Majtan

**Affiliations:** 1 Department of Cosmetic and Pharmaceutical Chemistry, Pomeranian Medical University in Szczecin, 70-111 Szczecin, Poland; anna.muzykiewicz@pum.edu.pl (A.M.-S.); lukasz.kucharski@pum.edu.pl (Ł.K.); 2 Department of Medical Chemistry, Pomeranian Medical University in Szczecin, 70-111 Szczecin, Poland; wojciech.zwierello@pum.edu.pl (W.Ż.); izabela.gutowska@pum.edu.pl (I.G.); 3 Department of Organic Chemical Technology and Polymer Materials, Faculty of Chemical Technology and Engineering, West Pomeranian University of Technology in Szczecin, 70-322 Szczecin, Poland; edyta.kucharska@zut.edu.pl; 4 Laboratory of Apidology and Apitherapy, Department of Microbial Genetics, Institute of Molecular Biology, Slovak Academy of Sciences, 845 51 Bratislava, Slovakia; jana.godocikova@savba.sk (J.G.); viktoriia.chirkova@savba.sk (V.C.); 5 Department of Physiology, Pomeranian Medical University in Szczecin, 70-111 Szczecin, Poland; katarzyna.piotrowska@pum.edu.pl; 6 Department of Human Nutrition and Metabolomics, Pomeranian Medical University in Szczecin, 71-460 Szczecin, Poland; karolina.jakubczyk@pum.edu.pl (K.J.); kinga.szymczykowska@pum.edu.pl (K.S.); 7 Department of Microbiology, Faculty of Medicine, Slovak Medical University, 833 03 Bratislava, Slovakia

**Keywords:** honey, phytochemicals, wound, antibacterial activity, skin, polyphenols

## Abstract

Medical-grade honeys (MGHs) are clinically applied therapeutic agents for wound management; however, their bioactive constituent profiles and biological performance in complex wound environments remain incompletely characterized. This study evaluated the wound-healing potential, antibacterial efficacy, and transdermal penetration capacity of three commercially available MGHs: manuka (MH), chestnut (ChH), and multifloral honey (MFH). MGHs were characterized for antioxidant capacity (DPPH and ABTS), total phenolic content (TPC), and phytochemical composition by HPLC and GC-MS. Wound healing activity was assessed using a scratch assay, transdermal penetration of phenolic acids through porcine skin using Franz diffusion cells, and antibacterial efficacy by MIC assay with and without artificial wound fluid exudate. H_2_O_2_ production was quantified after 24-h incubation. All MGHs demonstrated strong antioxidant activity and high TPC, with MH and ChH showing the highest values. MH exhibited the greatest polyphenol diversity, with gallic acid predominating (101.09 µg/g), and superior transdermal penetration attributable to elevated fatty acid content. Hydrophilic phenolic acids demonstrated rapid skin penetration within 3–5 h. Wound closure capacity in the scratch assay was honey type-dependent, with ChH showing the most favorable fibroblast migratory response. ChH also exhibited the highest antibacterial activity and H_2_O_2_ generation in wound fluid exudate. MGHs exhibit distinct, botanical origin-dependent biological activities, providing a rational framework for evidence-based MGH selection in clinical wound management.

## 1. Introduction

Honey represents a valuable natural product due to its multiple therapeutic benefits for humans. Although the outcomes of clinical studies on systemic honey use are inconsistent [1,2], evidence from topical application of honey-based products has conclusively demonstrated the therapeutic efficacy of honey in treating various wound types, including diabetic and obstetric wounds [3,4,5]. The wound-healing efficacy of topically applied honey has been evaluated in several recent meta-analyses [3,4,5]. An updated meta-analysis of eight randomised controlled trials encompassing 906 patients demonstrated that honey dressings significantly accelerated wound healing time by a mean of 17.13 days and increased the wound healing rate by 18.31% compared to control treatments in the treatment of chronic wounds [4]. In obstetric wounds, a meta-analysis of five RCTs including 353 patients found no statistically significant difference in final wound closure between honey and placebo groups, although honey significantly reduced pain intensity during treatment, highlighting that the clinical performance of honey is wound type-dependent and likely influenced by the biological activity of the specific honey product used [5].

A major limitation of conducted clinical studies is the lack of well-defined quality characteristics for the clinically tested honey, as well as its unknown biological activity, such as antibacterial, antioxidant, and anti-inflammatory properties. Therefore, for topical application, only well-characterized, sterile honey should be used. These honey-based products, classified as a medical device and approved for clinical application, with proven biological potential, are termed “medical-grade honey (MGH)” [6]. However, unlike manuka honey-based MGH, no other botanical honey types have been evaluated in robust clinical trials as registered MGH products, representing a significant gap in the translational evidence base.

Positive clinical outcomes from the topical application of MGH in wound care are robustly supported by in vitro findings that have comprehensively documented the antibacterial, antibiofilm, anti-inflammatory, and wound-healing activities of honey. Furthermore, multiple honey bioactive constituents (e.g., phenolic compounds, peptides) responsible for the observed activities have been identified. The high sugar content-mediated osmotic pressure of honey is considered a major antibacterial and wound healing mechanism of MGH due to regulatory frameworks classifying medical devices (as a physical barrier) [6]. However, the true therapeutic efficacy of MGH relies on multiple bioactive mechanisms, including modulation of immune and cutaneous cells, which positively impact the wound-healing process [7]. Honey can stimulate or inhibit the release of certain cytokines (TNF-α, IL-1β, IL-6) from human monocytes and macrophages, depending on the wound condition [7,8]. Additionally, it can reduce or increase neutrophil production of reactive oxygen species (ROS), depending on the wound microenvironment [9,10].

Beyond the high sugar content (about 80% of honey mass), which is a common feature of each honey, honey composition is highly variable in terms of phytochemicals that determine the biological properties of each honey type. For example, the antibacterial effect of manuka honey, the most extensively studied and clinically tested type of honey, is mediated by methylglyoxal (MGO) [11,12]. MGO accumulates in manuka honey by a conversion of its precursor, dihydroxyacetone, and its final concentration is a hundred-fold higher compared to other botanical honey types. This is a highly reactive molecule with antibacterial and anti-biofilm activities responsible for the structural modification and dysfunction of proteins and peptides in manuka honey [13,14].

In contrast to manuka honey and its reactive MGO, other honey botanical types, including chestnut and multifloral honeys, exhibit antibacterial effects primarily via hydrogen peroxide (H_2_O_2_) that accumulates in diluted honeys. However, the level of H_2_O_2_ varies significantly among honey types, and its final concentration depends on the presence of pollen catalase and/or certain phytochemicals [15]. Interestingly, H_2_O_2_ in diluted honey has been shown to act as a main mediator of honey regenerative effects on human keratinocytes [16]. The released H_2_O_2_ can subsequently cross the plasma membrane via a specific aquaporin channel. Honey exposure increases intracellular Ca^2+^ concentration through H_2_O_2_ production and redox regulation of Ca^2+^-permeable ion channels [16,17]. Another antibacterial compound, defensin-1, a bee-derived antibacterial peptide found in all types of honey, has been shown to induce cutaneous wound closure by enhancing keratinocyte migration and the matrix metalloproteinase-9 secretion. The dual antibacterial and immunomodulatory actions of both H_2_O_2_ and defensin-1 support the use of non-manuka MGHs in wound care.

Honey phytochemicals, in particular polyphenolic compounds such as flavonoids and phenolic acids, are minor constituents; however, they are the key factors that determine honey’s color, taste, and aroma. Furthermore, polyphenols exhibit strong antioxidant activities and potentiate the antibacterial effects of honey. We recently demonstrated that phenolic acids are readily released from honey-based hydrogels and accumulate in the skin, enabling subsequent successful penetration into deeper skin layers [18]. Polyphenolic compounds may exhibit diverse activities, such as antioxidant and free radical scavenging, modulation of inflammation, and stimulation of cellular migration and proliferation, in skin layers after effective penetration [19]. However, polyphenolic compounds permeate the skin significantly less effectively from pure honey than from honey-based hydrogels or emulsions [18]. Most MGHs used as wound care products are 100% honey with no other ingredients. In fact, a plethora of in vitro, in vivo, and clinical studies have analyzed pure honey, and the obtained outcomes served as the basis for the development of MGH.

From a clinical perspective, honey dressing reduced bacterial contamination of infected wounds and promoted wound healing more effectively than sugar dressing [20]. Due to the multifaceted action of honey in infected wounds, it remains unclear which honey constituents and/or factors exert the most pronounced effect on the wound healing process. Furthermore, the proteolytic environment of infected wounds can negatively affect the protein content of honey, including defensin-1 and the glucose oxidase (GOX) enzyme, which generates H_2_O_2_ in diluted honey.

Despite the extensive research on honey bioactivity, several critical knowledge gaps relevant to the clinical application of MGHs remain unaddressed. First, the transdermal penetration of phenolic acids from MGHs has not been systematically investigated. Second, the antibacterial efficacy of MGHs against wound pathogens has been mostly assessed under such in vitro conditions that do not reflect the complex biochemical environment of wounds. The effect of artificial wound fluid exudate on both antibacterial activity and H_2_O_2_ accumulation in MGHs of different botanical origins has not been comparatively evaluated. Third, while the fatty acid composition of honey is rarely reported, its potential role as an endogenous penetration enhancer, facilitating the transdermal delivery of hydrophilic phenolic compounds, represents a mechanistically novel concept. The present study addresses these gaps by integrating phytochemical profiling with transdermal delivery assessment and wound-environment-adapted antibacterial testing in three commercially registered MGHs. Specifically, we hypothesised that: (i) the fatty acid composition of MGHs would modulate the transdermal penetration of phenolic acids through disruption of the *stratum corneum* lipid architecture; (ii) the antibacterial efficacy of MGHs would be maintained or decreased in the wound fluid environment due to interactions between honey constituents and wound exudate components; and (iii) H_2_O_2_ accumulation capacity in wound fluid would differ significantly among MGH types, reflecting differences in GOX activity and phytochemical modulation.

## 2. Results

### 2.1. Antioxidant Activity of MGHs

The antioxidant activity and total polyphenolic content (TPC) of the analyzed MGHs are shown in Table 1. All analyzed MGHs exhibited antioxidative activity, as measured by the DPPH and ABTS methods. For the DPPH method, antioxidant activity ranged from 3.95 ± 0.46% for artificial honey (AH) to 86.74 ± 1.13% for manuka honey (MH). MH and chestnut honey (ChH) showed significantly higher free radical scavenging activity than MFH. Similarly, for the ABTS method, antioxidant activity ranged from 1.97 ± 1.36% for AH to 77.84 ± 1.07% for MH. Generally, MH showed the highest significant antioxidative activity (77.84 ± 1.07%) compared to ChH (69.11 ± 0.65%) and MFH (63.99 ± 1.19%).

An average TPC value ranged from 149.93 ± 6.38 mg/L for MFH to 358.37 ± 3.53 mg/L for ChH. TPC average values in MH and ChH were at a similar level of 332.56 ± 1.74 and 358.37 ± 3.53 mg/L, respectively, which was statistically higher compared to TPC in MFH. In the case of AF, as expected, no total polyphenol content was found (Table 1).

### 2.2. HPLC Analysis of MGHs

Table 2 presents the chemical composition of the analyzed MGHs, determined by HPLC. Twelve compounds belonging to the polyphenol family were identified in the analyzed MGHs, including 10 phenolic acids and 2 flavonoids. Among the phenolic acids, gallic acid, ellagic acid, ferulic acid, chlorogenic acid, vanillic acid, protocatechuic acid, gentistic acid, caffeic acid, *p*-hydroxybenzoic acid, and *m*-hydroxybenzoic acid were identified, while the flavonoids included rutin and quercetin. The analyzed MGHs showed varying levels of identified polyphenols (Figure S2). Gallic acid was the most abundant phenolic acid in MH, with an average concentration of 101.09 ± 5.82 µg/g. A significantly lower amount of gallic acid was found in ChH and MFH, with average amounts of 54.21 ± 2.20 and 8.53 ± 0.31 µg/g, respectively. MH also contained a higher content of ferulic acid compared to the other MGHs, while it was the only MGH containing chlorogenic acid, vanillic acid, and *p*-hydroxybenzoic acid. In the case of ChH, protocatechic acid was present in the highest amount (26.66 ± 2.40 µg/g) compared to the other MGHs, which had significantly lower levels of this compound (13.05 ± 0.91 and 14.21 ± 2.28 µg/g) for MH and ChH, respectively. Similarly, rutin was also present at the highest level in ChH (16.26 ± 0.95 µg/g), while in the other honeys it was 11.77 ± 1.62 µg/g in MH and 5.91 ± 0.51 µg/g in MFH. Generally, the lowest number of polyphenols was characterized in the MFH, while protocatechuic acid was the most abundant polyphenolic compound.

### 2.3. GC–MS Analysis of MGHs

The volatile and fatty components of MGHs determined by GC–MS are presented in Table S1. GC–MS analysis of the nonpolar fraction of MH revealed the presence of nonpolar compounds, primarily aliphatic and aromatic hydrocarbons and fatty acid derivatives. Long-chain alkanes, including tetradecane, eicosane, heneicosane, dotriacontane, and tetrapentacontane dominated among the identified substances. The chromatographic profile also revealed the presence of fatty acid esters (e.g., stearic acid methyl ester, tridecanoic acid methyl ester) and oleic acid amide (9-octadecenamide, Z). In the case of ChH, GC–MS analysis of the nonpolar fraction revealed the presence of nonpolar compounds, including aliphatic and aromatic hydrocarbons and fatty acid derivatives. Long-chain alkanes, such as eicosane, tetracosane, and heptadecane (3-methyl-), dominated among the identified substances. The chromatographic profile also revealed the presence of fatty acid amides (palmitoleamide) and esters (e.g., carbonic acid, decylpentadecyl ester), confirming the presence of a lipid fraction. In the case of MFH, GC–MS analysis of the nonpolar fraction revealed the presence of nonpolar compounds, primarily aliphatic and aromatic hydrocarbons and fatty acid derivatives. Long-chain alkanes, such as tetradecane, eicosane, heneicosane, and dotriacontane, are the predominant substances identified. Fatty acid derivatives were also identified, including oleic acid amide (9-octadecenamide, Z) and an ether (1,3-propanediol, ethyltetracosyl ether).

### 2.4. Mineral, Sugar, and Lactic Acid Content in MGHs

The content of selected minerals, vitamin C, and sugars in the analyzed MGHs is shown in Table S2. The vitamin C content in the analyzed MGHs ranged from 26.00 ± 0.00 to 75.67 ± 1.53 µg/g, with the significantly highest content found in ChH. AH was characterized by a very low vitamin C content, below 25 µg/g. ChH also contained the highest amounts of magnesium and calcium, 20.00 ± 0.00 and 32.00 ± 0.00, respectively, which were statistically higher than those in the other MGHs. All analyzed MGHs contained iron and chlorine at levels below 0.5 µg/g. MFH contained significantly less sugars, especially sucrose, followed by MH and ChH, 0.71 ± 0.02 mg/L and <0.25 for MH and ChH, respectively.

### 2.5. Cytotoxicity of MGHs

The viability of human dermal fibroblasts (BJ cell line) exposed to MGHs and AH at three concentrations (0.1%, 1%, and 10%) is presented in Figure S1. At the lowest concentration tested (0.1%), all MGHs and AHs maintained or slightly exceeded control cell viability, indicating no cytotoxicity at this concentration. At 1%, cell viability remained comparable to that of the untreated control across all tested MGHs, confirming biocompatibility within this range. At the highest concentration (10%), a statistically significant reduction in cell viability was observed for all MGHs compared to the control, indicating concentration-dependent cytotoxicity. Notably, AH at 10% did not reduce fibroblast viability compared to the control, demonstrating that the cytotoxic effect observed at this concentration for MGHs is attributable to their phytochemical constituents rather than their sugar content. According to ISO 10993-5:2009 [21], the standard for biological evaluation of medical devices, substances that reduce cell viability below 75% are considered cytotoxic. All MGHs maintained fibroblast viability above this threshold at clinically relevant concentrations of 0.1–1.0%, confirming their biocompatibility for wound care applications.

### 2.6. Wound Healing Properties of MGHs

**Figure 1:**
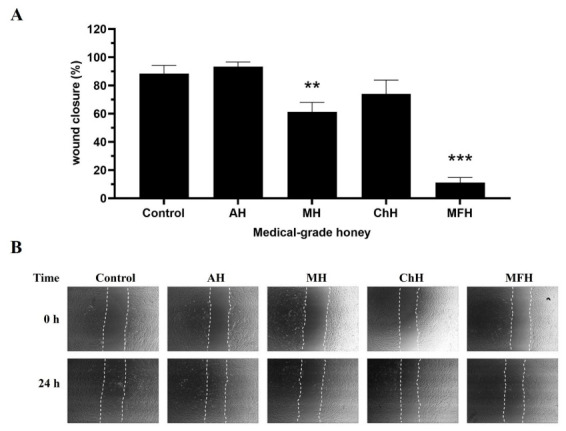
(**A**) Wound healing activity of medical-grade honeys (MGHs) and artificial honey (AH) at a concentration of 0.1%. Human dermal fibroblasts were used for the in vitro scratch assay. Data represent the mean with the standard deviation (SD) of the cell migration from three independent experiments. (**B**) Cells were incubated with AH and MGH dilutions for 24 h, with medium without additives serving as a control. Observations were performed at the time of wound creation (0 h) and after the 24-h incubation period (wound margin depicted as white dotted line). Asterisks indicate a significant difference *** *p* < 0.001, ** *p* < 0.01.

Figure 1 shows the results of the scratch assay performed on human dermal fibroblasts exposed to MGHs and AH at 0.1% concentration for 24 h. Among the MGHs tested, ChH showed the highest wound closure rate (73.97%), comparable to that of the untreated control. MH showed moderately reduced closure (61.27%), while MFH exhibited the lowest rate (11.17%). AH reached 93.30% wound closure, slightly exceeding the control. These results indicate that the wound-healing activity of MGHs at the tested concentration is honey type-dependent, with ChH performing most favourably among the three MGHs evaluated.

### 2.7. Transdermal Penetration Capacity of Selected MGH Polyphenols

The transdermal penetration of selected polyphenols from MGHs through porcine skin over 24 h, as measured using the Franz diffusion cell, is presented in Table 3. Additionally, the accumulation of polyphenols in the skin is presented in Table 4. During the 24-h skin penetration study, only some of the identified compounds penetrated the acceptor fluid, and this penetration was also dependent on the type of MGHs. The highest penetration rates were observed for MH, followed by ChH, and the lowest for MFH. In the case of MH, PaA penetrated most effectively, with a concentration of 48.17 ± 2.68 µg/cm^2^ in the acceptor fluid collected after 24 h. However, PaA penetration through the skin occurred only after 24 h. ChA also penetrated through the skin and accumulated in high amounts. The concentration of this compound in MH was 20.35 ± 0.85 µg/cm2 after 24 h of incubation. ChA was identified in the acceptor fluid already within 3 h of incubation. GA, out of all phenolic acids, penetrated through the skin most quickly, as documented by its presence in the acceptor fluid within 3 h of incubation with MH. However, its content after 24 h was 17.58 ± 2.16 µg/cm^2^, representing just over 2% of the compound’s penetration. In this honey, in the third hour of the study, other phenolic acids were also identified, including FA, ChA, and PrA. The compounds EA, VA, mA and Rut penetrated only after 24 h of testing, while CA did not penetrate into the acceptor fluid at all. A relatively high MH penetration of 10.39% was observed for EA (Table 3).

Analysis of the acceptor fluid collected after application of ChH to the skin revealed that significantly fewer compounds were able to penetrate, including some phenolic acids (EA, and FA), which penetrated within 8 h of incubation. However, PrA, GeA, and mA were identified only after 24 h of testing. The concentration of PrA, GeA, and mA in the acceptor fluid was 1.45 ± 0.32, 2.17 ± 0.60, and 11.51 ± 0.28, respectively. Flavonoids previously identified in ChH, such as Rut and Qe, did not penetrate at all within the 24-h incubation period (Table 3). In the case of MFH, a very low permeation of the analyzed compounds was demonstrated, except for some phenolic acids, such as EA, FA, PrA, and mA, which were identified only after 24 h of incubation. The highest permeation was observed at mA, with a concentration in the acceptor fluid of 11.25 ± 0.39 µg/cm^2^. Most of the polyphenols of MGHs accumulated in the skin, as determined by performing an extraction test on skin collected after 24 h immersion. In general, the highest concentration of the analyzed compounds was observed in the skin after MH application, whereas the lowest concentration was observed after MFH application. GA accumulated at the highest significant amount (77.50 ± 6.40 µg/g of skin) in the case of MH compared to the skin samples obtained after application of ChH and MH, with average concentrations of 7.83 ± 0.68 and 3.47 ± 0.30 µg/g of skin, respectively. Relatively high concentrations of other polyphenols were also identified in the skin samples after MH application. The highest accumulation percentage relative to honey applied to the skin was observed for Cha (21.70%), followed by Rut (14.35%), while the remaining samples ranged from 0.17 to 9.94%. When analyzing ChH in skin samples after its application, the highest actual accumulation of mA (53.28 ± 6.80 µg/g of skin) was observed compared to other honeys, which accounted for as much as 27.69% of the compound stored in the skin. The remaining values for the accumulated compounds ranged from 3.73 ± 1.21 µg/g of skin (2.54%) for GeA to 29.23 ± 1.94 µg/g of skin (8.72%) for Rut. The fewest compounds accumulated in the skin after MFH application and these were only GA, EA, FA, PrA and mA. The accumulation of these polyphenols ranged from 3.47 ± 0.30 µg/g of skin (1.87%) for GA to 31.43 ± 1.00 µg/g of skin (27.69%) for mA (Table 4).

**Figure 2:**
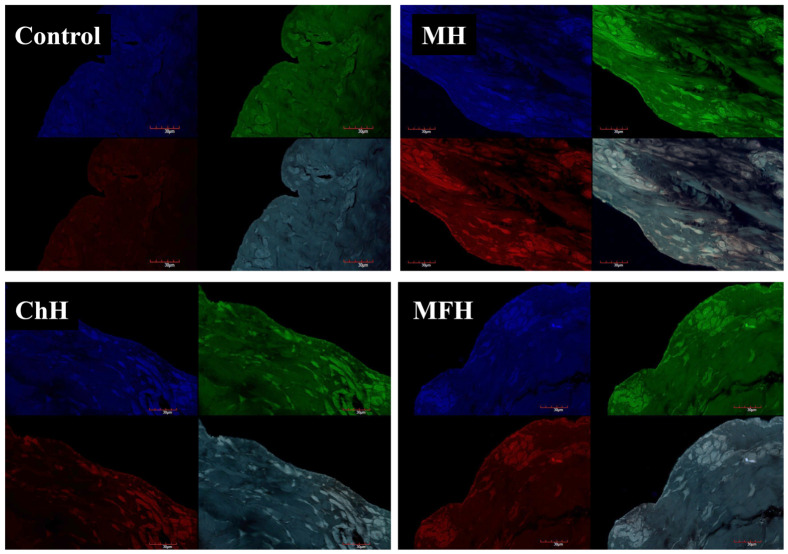
The microscopic photos of vertical slicing of porcine pig skin sections with medical-grade honeys (MGHs) and artificial honey (AH) after 24 h incubation. The polyphenols are visible in the upper layer of the skin, along the *stratum corneum*. The polyphenols are visualized under fluorescence (displayed in blue, red, and green colors). To observe autofluorescence across the entire light spectrum, slides were scanned using three separate lasers for three channels: channel 1: 405 nm (blue image; UV diode), channel 2: 488 nm (green image; blue laser), channel 3: 658 nm (red image; green laser), and channel 4: merged image. MH, manuka honey; ChH, chestnut honey; MFH, multifloral honey.

The highest deposition of polyphenols in skin samples after MH application was confirmed by cross-sectional skin photos, which are presented in Figure 2. It has been observed that polyphenols accumulate strongly, mainly along the SC of the skin. In images, marked with an intense fluorescence along with the *stratum corneum*. The polyphenols were visualized under a fluorescence microscope (blue, red, and green colors). Images obtained of the control pig skin treated with AH did not show any fluorescence (Figure 2).

### 2.8. Antibacterial Activity of MGHs Against Wound Pathogens in the Presence of Wound Fluid

**Figure 3:**
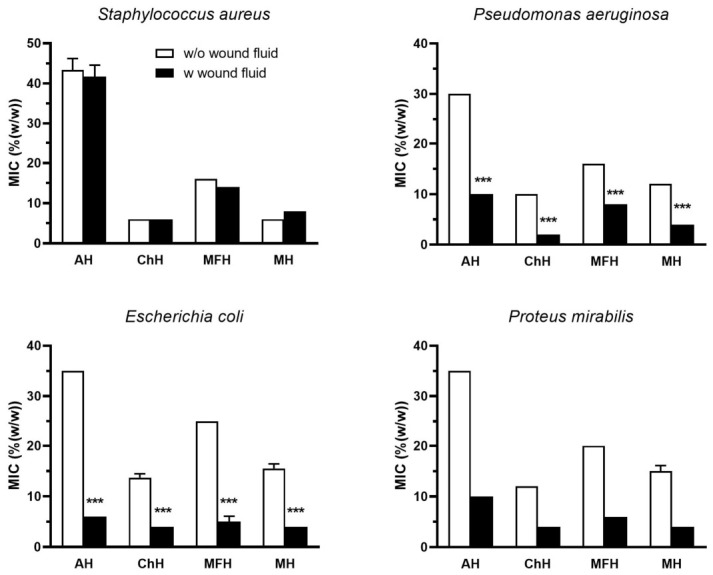
Antibacterial activity of medical-grade honeys (MGHs) and artificial honey (AH) against *Staphylococcus aureus*, *Pseudomonas aeruginosa*, *Escherichia coli*, and *Proteus mirabilis* in the absence or presence of artificial wound fluid exudate was determined by a minimum inhibitory concentration (MIC) assay. The data are expressed as mean MIC values with standard deviation (SD). Differences between MGH and wound fluid exudate-enriched MGH were analyzed by the Mann–Whitney test. Asterisks indicate a significant difference *** *p* < 0.001. MH, manuka honey; ChH, chestnut honey; MFH, multifloral honey.

The antibacterial activity of MGHs against selected wound pathogens, *S. aureus*, *P. aeruginosa*, *E. coli*, and *P. mirabilis*, in the absence or presence of wound fluid exudate at a final concentration of 20%, was expressed as a MIC value determined by the microdilution assay. The MIC values of MGHs and AH against all bacteria are shown in Figure 3. The mean MIC values of AH ranged from 30% to 43% for *P. aeruginosa* and *S. aureus*, respectively. Among the tested MGHs, ChH showed the highest antibacterial efficacy with MIC values of 6%, 10%, 14%, and 12% against *S. aureus*, *P. aeruginosa*, *E. coli,* and *P. mirabilis*, respectively, followed by MH and MFH. The antibacterial efficacy of MGHs in the presence of wound fluid exudate was augmented against *P. aeruginosa*, *E. coli,* and *P. mirabilis*, but was unchanged in the case of *S. aureus*. Interestingly, wound exudate significantly increased the antibacterial activity of AH against *P. aeruginosa*, *E. coli*, and *P. mirabilis* by 3.0-, 5.8-, and 3.5-fold, respectively (Figure 3). Wound fluid exudate at 20% concentration did not exhibit antibacterial activity against any of the bacteria.

### 2.9. H_2_O_2_ Accumulation in MGHs in the Presence of Wound Fluid

**Figure 4:**
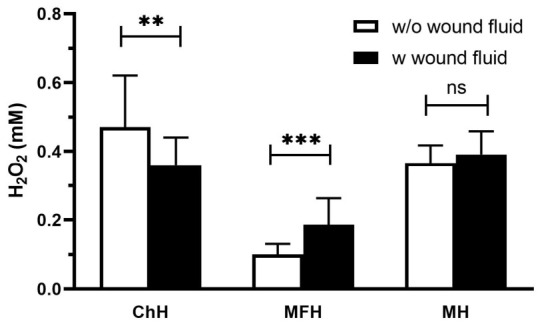
Determination of hydrogen peroxide (H_2_O_2_) content in diluted medical-grade honeys (MGHs) after 24-h incubation with or without artificial wound fluid exudate. Differences between MGHs and MGHs enriched with wound fluid exudate were analyzed by an unpaired *t*-test. Asterisks indicate a significant difference. The data are expressed as mean values with SD. *** *p* < 0.001, ** *p* < 0.01; ns, non-significant.

H_2_O_2_ generation in diluted honey is responsible for its antibacterial activity, and increasing its concentration could further enhance it. Surprisingly, MH contained a higher amount of H_2_O_2_ compared to the MFH sample (Figure 4). The highest level of H_2_O_2_ was documented in ChH sample. Despite the artificial wound exudate causing an increase in the antibacterial effect of all AH and MGHs against each bacterium except for *S. aureus*, a statistically significant enhancement of H_2_O_2_ content (*p* < 0.001) was shown only in MFH. On the other hand, no change in H_2_O_2_ level occurred in exudate-enriched MH, and a significant decrease in H_2_O_2_ content (*p* < 0.01) was observed in ChH supplemented with exudate.

## 3. Discussion

MGHs have gained significant interest in wound management, primarily due to the absence of risk of bacterial resistance with long-term use and their role as ideal wound-healing agents, maintaining a moist wound environment throughout the entire healing process.

In this study, we comprehensively characterized the biological properties of three MGHs, based on different types of 100% honey, including manuka honey, chestnut honey, and multifloral honey, which are registered on the pharmaceutical market as medical devices. As a control, artificial honey (sugars only) was used in the study. We found significant differences among MGHs in various investigated parameters and properties. The botanical origin and phytochemical composition were the primary factors responsible for the observed differences, which determined their antioxidant, antibacterial, and wound-healing properties.

Honey phytochemicals, such as polyphenolic compounds, despite their low concentrations, significantly contribute to the antioxidant and anti-inflammatory effects of honey in wound healing [22]. During wound healing, ROS are widely generated at each stage, and their levels become elevated, which is considered a substantial factor in the delay in wound healing [23]. Oxidative damage caused by excessive ROS is a common problem associated with chronic, difficult-to-heal wounds. Honey polyphenols exhibit free radical scavenging ability and antioxidant activity, as also demonstrated in this study. Manuka honey showed the greatest antioxidant activities compared to chestnut and multifloral medical-grade honeys. Similarly, MH and ChH contained the highest and comparable TPC, which was twice that of MFH. Numerous studies have provided compelling evidence that manuka honey with high MGO content exhibits higher antioxidant activity and higher average TPC values than other botanical types of honey [24,25,26,27]. Due to its bioactive properties, manuka honey has often been used as a “gold standard” for assessing and comparing the antioxidant properties of other honey types [28]. On the other hand, several studies reported that buckwheat and honeydew honeys showed antioxidant potential comparable to, or even superior to, that of manuka honey [29,30]. The high polyphenolic content in honey is associated with greater antibacterial activity, as polyphenols can enhance and strengthen honey’s antibacterial properties. Therefore, buckwheat or honeydew honey, rich in polyphenols, exhibits a strong antibacterial effect and could serve as a suitable honey source for new medical-grade honey used in wound care.

The diversity of polyphenols in honey is more important than their total amount, as it can be used as a tool for botanical and geographical discrimination. In the present study, MH was the richest MGH in terms of polyphenol diversity. The most abundant phenolic acid in MH was gallic acid. The high content of gallic acid in manuka honey was also documented in a study by Afrin et al. (2018) [31], who reported that gallic acid constituted primarily 49% of the total phenolic acid content among all phenolic acids identified in manuka honey. Similarly, a very high content of gallic acid (23% of total phenolic acids), which was the dominant compound in manuka honey, was also confirmed by Yao et al. (2013), suggesting gallic acid as a marker (along with abscisic acid) for authenticating manuka honey samples [32]. Gallic acid has numerous beneficial effects, including its role in wound healing. It stimulates the expression of antioxidant genes and affects wound healing under both normal and hyperglycemic conditions, thereby mimicking diabetes by accelerating the migration of keratinocytes and fibroblasts [33]. Furthermore, gallic acid activates factors characteristic of wound healing, including focal adhesion kinase (FAK), c-Jun N-terminal kinase (JNK), and extracellular signal-regulated kinase (ERK). In addition, collagen- and hyaluronic acid-based hydrogels with dopamine and gallic acid as the main antioxidant component demonstrated positive healing benefits, resulting in faster cell proliferation and migration [34]. The authors highlight gallic acid as the primary polyphenolic compound and attribute its effect on wound healing to its high antioxidant activity.

To successfully register honey as an MGH, it is necessary to evaluate its cytotoxicity, as natural products can contain substances that are harmful to cells [35]. In general, honey is considered safe and low-toxicity [6]. According to the ISO 10993-5:2009 standard “Biological evaluation of medical devices” substances are considered biocompatible if they do not reduce cell viability below 75% [35]. In the present study, MGHs at concentrations of 0.1–1.0% were found to be non-toxic and suitable for biomedical applications. The lower concentrations of all MGHs and AHs analyzed generally increased cell viability after 24 h of incubation. The highest cell viability was observed in the control group at 0.1% AH, MH, and MFH concentrations. Interestingly, cell viability at 0.1% was even slightly elevated above control levels for MH, MFH, and AH, suggesting a mild proliferative or cytoprotective stimulus at low concentrations. Slightly lower cell viability, similar to that of the control, was also observed at 1% AH, MH, and MFH concentrations. On the other hand, the lowest cell viability among all MGHs analyzed was observed at 10%; however, in the case of ChH, it remained above 70%. Similarly, the highest cell viability of human fibroblasts was observed with Kalulut honey at the lowest concentration, specifically 3.125 mg/mL [36]. Furthermore, other studies confirmed varying cytotoxicity toward cutaneous cells, including keratinocytes and fibroblasts, which was most often dependent on honey concentration. In our previous study [18], cell viability and morphology did not differ from those of untreated cells when different natural honeys (heather, linden, and buckwheat) were used at a concentration of 1%, and in the case of rapeseed honey, at a concentration of 2.5%. Honey at a concentration of 5% showed cell viability ranging from 20% to 66%. Taken together, the outcomes of the honey cytotoxicity test depend primarily on the honey’s phytochemical composition, the type of cells (e.g., keratinocytes, fibroblasts), and the concentrations of honey tested. Furthermore, the concentration-dependent cytotoxicity observed at 10% for all MGHs, but not for AH, clearly implicates the phytochemical fraction rather than osmotic sugar effects as the primary cytotoxic driver at higher concentrations.

The in vitro scratch assay is a valuable technique for analyzing the molecular and cellular mechanisms of cell migration and evaluating therapeutic compounds before clinical use [37]. Cell proliferation and cell migration to the site of tissue injury are essential for effective wound healing. Based on the cytotoxicity results, the wound healing activity of MGHs in this study was determined at a concentration of 0.1%. At this concentration, ChH supported wound closure at a rate comparable to the untreated control, while MH and MFH showed reduced fibroblast migration. These findings indicate a concentration- and composition-dependent effect of MGHs on fibroblast behavior rather than an intrinsic lack of wound-healing potential. These results contrast with those of other studies [38,39,40] that used a similar concentration of the tested honey. It was found that migration of HaCaT keratinocyte cells increased with increasing honey dilutions (0.01%, 0.1%, and 1%). The highest wound closure rate was achieved at 0.1% honey dilution. In a study by Ranzato et al. (2012) [38], keratinocytes (HaCaT cells) exposed to three different honeys (acacia, manuka, and buckwheat) at a concentration of 0.1% exhibited significantly higher wound closure rates at 24 h compared to the control (untreated cells), with similar effects observed among the honeys. Identical results were obtained when the human fibroblast cell line (46BR.1N) was used instead of keratinocytes [39]. Observed discrepancies between our results and the published results might, at least in part, be due to differences in methodological approach. During the wound healing assay, the concentration of FBS in the conditioned culture medium was reduced to 1% in our study to minimize cell proliferation. Controversially, complete culture medium (10% FBS) was not modified in wound healing assays in other studies. Moreover, MH and ChH, used in our study, are rich in phytochemicals, some of which can also induce oxidative stress or inhibit fibroblast proliferation, potentially leading to slower cell migration and reduced wound closure. In fact, high content of fatty acid derivatives and volatile compounds in MG and MFH, determined in this study by GC-MS, may contribute to the inhibition of cell proliferation. The mechanism of action is likely to include increased oxidative stress, membrane disruption, induction of apoptosis, and potential pro-inflammatory effects [40]. Among MGHs, ChH exhibited significantly higher content of vitamin C, calcium, and magnesium. Magnesium ions enhanced cell migration by activating the MEK/ERK pathway [41]. In other studies, treatment of epidermal keratinocytes with magnesium ions increased hyaluronic acid synthase expression via glycogen synthase kinase 3 and cyclic AMP response element-binding protein, leading to increased hyaluronic acid production and promoting wound healing [42]. Finally, it is essential to note that the in vitro model employed does not fully replicate the complexity of the in vivo wound healing process. The wound healing assay primarily assesses rates of fibroblast migration and proliferation, without considering interactions with immune cells or the presence of microorganisms.

The high MGO content characteristic of MH warrants a more nuanced interpretation in the context of wound healing. MGO is the primary driver of antibacterial activity of MH, but it is also a highly reactive dicarbonyl compound capable of glycating proteins and peptides, forming advanced glycation end-products (AGEs) that may impair cellular function and delay tissue regeneration [43]. This is particularly relevant in diabetic wound environments, where endogenous MGO accumulation already contributes to glycation-mediated tissue damage. Furthermore, we showed that MGO in MH negatively impacts the enzymatic and antibacterial activities of GOX and defensin-1, respectively [13,14]. In addition, it may exert concentration-dependent cytostatic effects on fibroblasts and keratinocytes [44,45]. The reduced fibroblast migration observed in the scratch assay for MH in the present study may partly reflect such effects at the tested concentration. These considerations do not diminish the clinical value of MH-based MGH, but underscore the importance of concentration optimisation and careful patient selection—particularly in diabetic wound care—when choosing between MGH types. Therefore, when assessing the therapeutic potential of MH, both the beneficial and potentially harmful effects of MGO should be considered. MGO concentration was not quantified in this study, so the observed biological properties are likely to be due to the combined effects of multiple honey components, including polyphenols, organic acids, sugars, enzymes, minerals, and other secondary metabolites.

MGH is primarily considered an antibacterial agent that eliminates wound infections and bacterial biofilm in the wound. It contains more than 200 bioactive constituents that can affect different processes in immune and cutaneous cells within the wound. Skin penetration studies are crucial when analyzing new cosmetic or dermatological preparations applied to the skin. The active substances can penetrate through the *stratum corneum* into deeper layers of the skin. The penetration of honey’s secondary metabolites plays a crucial role because their location in the deeper layers of the skin allows them to exert their biological effects, such as antioxidant or anti-inflammatory effects. In the present study, 24-h skin penetration of MGHs active substances was examined. Only a few of the identified compounds penetrated the acceptor fluid, which was also dependent on the type of MGH. Generally, the highest penetration was observed for MH, followed by ChH, and the lowest for MFH. There are only a few reports characterizing the penetration of phenolic acids from different types of honey [18,46,47]. Different penetration profiles of phenolic acids were observed in our study, depending on the type of honey. The highest percentage of phenolic acids penetrated the skin was observed with MH, with the highest levels for chlorogenic acid and *p*-hydroxybenzoic acid. The differences in penetration profiles among MGHs are primarily due to the diverse composition of each MGH. A high fatty acid content in MH may increase the penetration of active constituents through the *stratum corneum* into deeper layers. Fatty acids interfere with the lipid structure of the *stratum corneum* and loosen the “lipid cement”, which is the main protective barrier of the skin. Furthermore, fatty acids are incorporated into the *stratum corneum* lipids, thereby increasing the lipid layer’s fluidity [48,49]. Additionally, the lipophilicity of honey’s active compounds plays a crucial role in their penetration through the skin. Zilius et al. (2016) [50] investigated the penetration of phenolic acids from cosmetic formulations containing propolis, suggesting that lipophilicity is the primary factor influencing their penetration. This means that more hydrophilic compounds are released and penetrate the *stratum corneum* lipid layer more effectively. The authors found that caffeic acid was released and penetrated the skin in lower amounts compared to vanillic acid, which has lower lipophilicity. This is confirmed by our results, in which GA, ChA, and GeA, characterized by low lipophilicity (GA − log P 0.7; ChA − log P 0.7 and GeA − log P 0.42), penetrated most rapidly, already within 3 h after application to the skin in the case of GA and GeA, and within 5 h in the case of ChA. In comparison, CA, characterized by higher lipophilicity (CA − log P − 1.5), did not penetrate at all. Taken together, these findings support our hypothesis that fatty acid composition functions as an endogenous penetration enhancer in MGHs, providing a mechanistic basis for the observed differences in phenolic acid skin bioavailability across botanical types.

The wound environment can modulate the action of wound dressings and their active substances. Many of the causative organisms of wound infections can produce bacterial proteases as virulence factors to overcome host defenses [51]. Additionally, other wound fluid substances can affect the honey’s active compounds. In our previous work [52,53], we demonstrated that GOX and defensin-1 are proteinase-sensitive molecules; however, the overall antibacterial effect of proteinase K-treated honey samples did not change significantly. In the present work, artificial acute wound fluid exudate had a positive effect on the antibacterial activity of MGHs against the most tested bacteria, except for *S. aureus*. Likely, wound fluid acted synergistically with the high sugar content of MGHs, as antibacterial activity also increased in AH supplemented with wound fluid. *S. aureus* is a model bacterium for commercially available testing of honey antibacterial potential due to its highest prevalence in infected wounds and the fact that it is not sensitive to honey’s high sugar content in comparison to other bacterial strains (e.g., *P. aeruginosa*). ChH exhibited the highest antibacterial activity and produced the highest H_2_O_2_ content among the MGHs tested in the study. Surprisingly, diluted MH accumulated a higher level of H_2_O_2_ than the MFH sample, indicating thermal processing of MFH during its production.

A few limitations must be acknowledged in the present study. The first limitation is the absence of quantitative MGO determination in the MH sample. Although MGO is discussed as a key bioactive constituent, its concentration was not directly measured, which creates a disconnect between the mechanistic interpretation and the experimental data. In the present study, a certified commercial MH product with a unique manuka factor (UMF) of at least 15+ was used. Second, the phytochemical profiling in this study was intentionally focused on phenolic acids and flavonoids, as these are the predominant skin-penetrating bioactive compounds relevant to the transdermal delivery objective of the study. However, honey contains additional bioactive constituents not assessed here, including bee-derived proteins and peptides (e.g., defensin-1, glucose oxidase, apisimin, MRJPs) and polyamines and polyamine-derived phenolamides (e.g., spermidine-, putrescine-, and spermine-conjugates reported in pollen and honey. Despite the negligible polyamine concentration in honey [54], these compounds may contribute to the observed biological activities, and their exclusion limits the comprehensiveness of the chemical profiling.

## 4. Materials and Methods

### 4.1. MGH Wound Care Products

Three certified honey-based wound care products, representing the principal botanical types among those commercially available, containing 100% gamma-irradiated honey of different botanical origins, namely manuka honey (MH; LOT Number: WO034248), chestnut honey (ChH; LOT number: 2403921), and multifloral honey (MFH; LOT number: EGN), were used in the study. As a negative control, artificial honey (AH) was prepared by dissolving 39 g D-fructose, 31 g D-glucose, 8 g maltose, 3 g sucrose, and 19 g distilled water and stored in darkness at 2–5 °C. The samples were placed in a plant material storage room at the Department of Cosmetic and Pharmaceutical Chemistry of the Pomeranian Medical University (no. H-AM2025-03). The honey samples were stored in accordance with the Polish Standard PN-88/A-77626 [55]. The storage temperature did not exceed 18 °C. The samples were stored in commercial packaging, protected from light. All chemicals were purchased from Sigma-Aldrich (Darmstadt, Germany), unless otherwise stated.

### 4.2. Identification and Quantification of Phenolic Acids in MGHs

For HPLC analyses, MGH samples were diluted 1:5 with distilled water (25 °C) and mixed until complete dissolution. The resulting solutions were mixed with a magnetic stirrer for 10 min and then centrifuged. Quantitative analyses of polyphenol content in the analyzed honeys and acceptor fluids collected for the skin permeation study and after skin extraction were performed using an HPLC system (Knauer, Berlin, Germany) equipped with a WellChrom K-2600 UV detector and a Hypersil ODS C18 column (125 × 4 mm, particle size 5 µm). Compounds were identified using previously described methods [56,57] with a 1% aqueous acetic acid solution (eluent A) and methanol (eluent B) as the mobile phase. Elution was performed with a gradient: 90% A/10% B (0–6 min), 84% A/16% B (7–25 min), 72% A/28% B (26–37 min), 65% A/35% B (38–47 min), 50% A/50% B (48–64 min), followed by re-equilibration to the initial conditions (90% A/10% B, 65–70 min). The injection volume of the analyzed sample was 20 µL. All samples were injected in triplicate.

### 4.3. Gas Chromatography Coupled with Mass Spectrometry (GC–MS)

Characterization of the nonpolar compound fraction in MGH samples was performed using gas chromatography–mass spectrometry (GC–MS). Analysis was performed using a GC–MS-QP2020 NX instrument (Shimadzu, San Jose, CA, USA) equipped with a Shimadzu SH-I-5MS capillary column measuring 30 m × 0.25 mm with a 0.25 µm film thickness. Initially, the column temperature was maintained at 40 °C for 2 min, then increased linearly to 300 °C at 10 °C/min, followed by a final temperature hold for 2 min. The sample injection volume was 1000 µL. Component identification was performed by comparing the obtained mass spectra with reference data contained in the NIST-2020 library. Each sample was analyzed three times.

Compound identification was performed by comparing mass spectra with the NIST database and was supported by literature retention indices (RI). The RI values were taken from the NIST Chemistry WebBook, and the concept of linear retention indices is based on the method of van den Dool and Kratz [58,59].

### 4.4. Determination of Mineral, Sugar, and Lactic Acid Contents

An RQflex^®^ 20 refractometer (Reflequant, Supelco, Inc., Bellefonte, PA, USA) was used to determine the content of sucrose, total sugars (including glucose and fructose), lactic acid, vitamin C, and selected minerals. The analysis was performed by adding 0.3 mL of the test extract and a control sample. The minimum detectable concentrations for magnesium, iron, calcium, chloride, and malic acid were 5 mg/L. For vitamin C, the limit was 25 mg/L; for total sugars, 65–650 mg/L; and for sucrose, 1–100 mg/L.

### 4.5. Determination of Antioxidant Activity (DPPH, ABTS) and Total Phenolic Content (TPC) of MGHs

Analysis of antioxidant activity and total phenolic content was performed using a U-5100 UV–Vis spectrophotometer (Hitachi, Tokyo, Japan) [18]. Briefly, for DPPH, a 0.15 mL aliquot of each honey tested (MGH) was combined with 2.85 mL of 0.3 mM DPPH solution dissolved in 96% (*v*/*v*) ethanol. The incubation time for the analyzed sample was 10 min in the dark at room temperature. Analysis was performed at 571 nm. For ABTS radical scavenging evaluation, a stock solution was prepared by dissolving 7 mM ABTS in 2.45 mM aqueous potassium persulfate. Then, 2.5 mL of the ABTS solution was mixed with 0.025 mL of the honey sample (MGH) to be analyzed. The incubation time was 6 min, and absorbance was measured at 734 nm. The results of both tests (DPPH and ABTS) were expressed as a percentage of radical inhibition and calculated according to the following Equation (1):

%DPPH (ABTS) scavenging = 1 − As/Ac × 100%(1)

where:

As = absorbance of the tested sample

Ac = absorbance of the control sample

Measurements were carried out in triplicate for each MGH.

The results were also expressed as Trolox equivalents, a standard antioxidant, with activity reported as mg TE/g of honey.

Total phenolic content (TPC) in MGH was determined according to the Folin–Ciocalteu method [52]. 0.15 mL of the sample was used for the reaction, which was combined with 0.15 mL of Folin–Ciocalteu reagent, 1.35 mL of 0.01 M sodium carbonate solution, and 1.35 mL of distilled water. The incubation time was 15 min. Absorbance measurements were made at 765 nm. Gallic acid was used to prepare a calibration curve. Each determination was repeated three times independently.

### 4.6. Determination of H_2_O_2_ Content in Diluted MGHs

The H_2_O_2_ content in the MGHs was determined using a Megazyme GOX assay kit (Megazyme International Ireland Ltd., Bray, Ireland) according to the manufacturer’s instructions. Forty percent (*w*/*w*) of the MGH solutions in 0.1 M potassium phosphate buffer (pH 7.0) or artificial wound fluid (pH 7.4) were prepared. After 24 h of incubation at 37 °C, each MGH and the H_2_O_2_ standard were tested in duplicate in a 96-well microplate. Absorbance was measured at 510 nm using a Synergy HT microplate reader (BioTek Instruments, Winooski, VT, USA).

### 4.7. Determination of Antibacterial Activity of MGHs

The bacterial isolates *Staphylococcus aureus* CCM4223, and *Pseudomonas aeruginosa* CCM1960 were obtained from the Czech Collection of Microorganisms (Brno, Czech Republic) and *Escherichia coli* ZPM90 and *Proteus mirabilis* ZPM82 were acquired from the Department of Medical Microbiology at the Slovak Medical University (Bratislava, Slovakia).

The antibacterial efficacy of the MGHs against bacterial pathogens was evaluated with a minimum inhibitory concentration (MIC) assay as described by Bucekova et al. (2023) [53]. Briefly, an overnight bacterial culture was adjusted to 10^8^ colony-forming units (CFU)/mL and diluted in Mueller–Hinton broth (MHB) (Oxoid, Basingstoke, UK) to a final concentration of 10^6^ CFU/mL. Then, 10-μL aliquots of suspension were inoculated into each well of sterile 96-well polystyrene U-shaped plates (Sarstedt, Germany). The final volume in each well was 100 μL, consisting of 90 μL of sterile medium or diluted honey and 10 μL of bacterial suspension. After 18 h of incubation at 37 °C, bacterial growth inhibition was determined by visual inspection. The MIC was defined as the lowest concentration of honey inhibiting bacterial growth. All tests were performed in triplicate and repeated three times. Serial dilutions of each honey sample were prepared from a 50% (*w*/*v*) honey solution, resulting in final concentrations ranging from 45% to 4%.

The antibacterial activity of MGHs was also evaluated in the presence of artificial wound fluid exudate (BioChemazone, Edmonton, AB, Canada). The final concentration of wound fluid exudate in each honey dilution was 20%. MIC was determined as stated above.

### 4.8. Determination of MGHs Cytotoxicity

Fibroblast viability (MTT) was assessed after exposure to AH and MGHs. Glucose solutions and three MGHs (MH, ChH, and MFH) were used to assess cytotoxicity. Preparations were prepared in complete culture medium at concentrations of 0.1%, 1%, and 10% (*v*/*v*) and then filtered through sterile 0.22 µm filters. Human dermal fibroblasts (BJ) (ATCC CRL-2522) were used to assess cytotoxicity. Cells were cultured in DMEM supplemented with 10% FBS, antibiotics, and L-glutamine at 5% CO_2_, 37 °C, and 95% humidity. Cells were seeded in 96-well plates at 5000 cells per well. After 24 h, the medium was replaced with glucose solutions or prepared dilutions of MGHs (0.1%, 1%, and 10%) and incubated for 72 h. Absorbance was measured at 570 nm. Results were expressed as a percentage of cell viability relative to the negative control. Each experimental variant was performed in three independent biological replicates, each repeated three times in parallel.

### 4.9. Wound Healing Assay

The effect of AH and MGHs on fibroblast migration was examined using a scratch assay. The human BJ cell line ATCC CRL-2522 (LGC Standards Sp. z o.o., Łomianki, Poland) was used for analysis. Cells were seeded in 24-well plates and cultured until a compact monolayer formed. Controlled dissection of the monolayer, creating a “linear wound,” was performed after reaching 80–90% confluence. Detached cells were removed by repeated washes with PBS, and 1% FBS was added to the medium to minimize proliferation. A single concentration of AH and MGH (0.1%) was used for the study, allowing for the observation of migration with minimal cytotoxicity. The preparations were prepared in culture medium and filtered through a 0.22 µm filter, following the procedure used in the cytotoxicity assay. Cells were incubated in AH and MGH solutions for 24 h, with medium without additives serving as a control. Images of the wound were recorded immediately after its formation (0 h) and after 24 h of incubation using a Zeiss PrimoVert microscope under brightfield illumination (10×). Analysis of the gap width was performed using ZEISS ZEN 3.1 software (Zeiss, Oberkochen, Germany). Fibroblast migration was expressed as a percentage of wound closure relative to the initial value. Each experimental variant was performed in triplicate within three independent biological runs.

### 4.10. Skin Permeation Studies

The study of the permeation of selected phenolic acids and flavonoids through porcine skin was performed using Franz diffusion cells (SES GmbH Analyse Systeme, Bechenheim, Germany) with a thermostat (VEB MLW Prüfgeräte-Werk type 3280, Leipzig, Germany) maintaining a constant temperature of 37.0 ± 0.5 °C in acceptor chambers. The diffusion surface area was 1 cm^2^, and the volumes of the donor and acceptor chambers were 2 mL and 8 mL, respectively. The acceptor fluid was PBS buffer (pH 7.4). Porcine skin, which closely resembles human skin, was placed between the donor and acceptor chambers [60,61]. Before the permeation studies were initiated, skin integrity was assessed by measuring its impedance with an LCR 4080 m (Voltcraft, Conrad Electronic, Germany) in parallel mode at 120 Hz (measurement error for values in the kΩ range < 0.5%) [62,63]. One gram of each MGH was applied to the donor chamber. The penetration test lasted 24 h, and 0.3 mL of acceptor fluid was collected at 3, 5, 8, and 24 h. Each acceptor chamber was replenished with fresh PBS buffer after collection. The concentration of selected phenolic acids and flavonoids in the acceptor phase was determined by HPLC. The cumulative mass of penetrating compounds was expressed in µg.

The accumulation of selected compounds in the skin after 24 h was assessed using a modified method described by Ossowicz-Rupniewska et al. [63]. After completing the permeation study and dismantling the diffusion cells, each skin sample was gently rinsed with PBS buffer (pH 7.4), and a 1 cm^2^ fragment corresponding to the diffusion area was excised. After incubation of the skin samples in concentrated methanol for 24 h, the samples were homogenized using a homogenizer (IKA^®^ T18 digital ULTRA TURRAX, Germany) and then centrifuged at 3500 rpm for 5 min. The amount of accumulated active compounds in the skin was expressed as µg/g of skin.

### 4.11. Fluorescent Microscopy

Skin samples collected from the Franz diffusion cell after the permeation experiment were fixed in 4% buffered paraformaldehyde for 24 h. Skin samples were then dehydrated in various concentrations of alcohol and xylene and embedded in paraffin blocks. The paraffin blocks were cut into 5 μm slices using a rotary microtome. After mounting on histopathology slides, the sections were rehydrated and dried. Neu’s reagent (1% 2-aminoethyldiphenylborate in methanol) was used to visualize the polyphenol content [64]. Sections were scanned using a confocal microscope (FV-1000, Olympus, Tokyo, Japan) with a 405 nm diode laser, an Olympus IX81 inverted microscope, and FV10-ASW 4.2 software (Olympus).

### 4.12. Statistical Analysis

Results are presented as the mean ± standard deviation (SD) and the relative standard deviation (RSD). One-way analysis of variance (ANOVA) was performed to assess the data, and Tukey’s test was used to evaluate the significance of differences between individual MGHs (α = 0.05). The Mann–Whitney test and *t*-test were used to compare the differences in antibacterial effect and the H_2_O_2_ content between MGH and MGH incubated with wound fluid exudate, respectively. Data with *p*-values smaller than 0.05 were considered statistically significant. All statistical analyses were performed using either Statistica 13 PL software (StatSoft, Krakow, Poland) or GraphPad Prism 11.0.0 (GraphPad Software Inc., La Jolla, CA, USA).

## 5. Conclusions

This comprehensive study demonstrates that MGHs exhibit distinct biological activities, primarily determined by their botanical origin and phytochemical composition. All three MGHs investigated displayed substantial antioxidant capacity and TPC, with significant inter-honey variations in specific bioactive constituents. MH demonstrated the highest polyphenol diversity and superior transdermal penetration of phenolic acids, attributable to its elevated fatty acid content, facilitating enhanced permeation through the *stratum corneum*. ChH exhibited the most pronounced antibacterial efficacy and H_2_O_2_ generation capacity in the presence of wound fluid exudate. Cytotoxicity evaluation confirmed the biocompatibility of all MGHs at clinically relevant concentrations (0.1–1.0%), meeting ISO 10993-5:2009 standards. Transdermal penetration analysis revealed that phenolic acids successfully accumulate in skin layers, with hydrophilic compounds such as gallic acid, chlorogenic acid, and gentisic acid demonstrating rapid penetration within 3–5 h post-application. Artificial wound fluid exudate enhanced antimicrobial activity against most tested pathogens, suggesting synergistic interactions between honey constituents and components of the wound environment. The concentration- and composition-dependent effects observed in the scratch assay underscore the complexity of MGH–cell interactions in vitro and highlight the importance of selecting appropriate honey types and concentration ranges for specific wound care applications. The identification of diverse nonpolar compounds by GC–MS, including beeswax-derived lipids and fatty acid esters, provides insights into differential skin-penetration capacities among MGHs. These lipophilic constituents may function as natural penetration enhancers by disrupting the *stratum corneum* lipid architecture, thereby facilitating the transdermal delivery of hydrophilic bioactive compounds. This study provides robust evidence that MGHs represent efficacious therapeutic agents for wound management, with biological activities fundamentally governed by botanical origin and phytochemical composition. The demonstrated antibacterial efficacy, antioxidant capacity, and ability to deliver bioactive phenolic compounds to skin tissues support the continued clinical application of MGHs. Future investigations should focus on optimizing MGH concentrations for specific wound types and conducting controlled clinical trials to validate in vitro findings. The distinct biological profiles suggest that botanical origin should be carefully considered when selecting MGHs for specific wound care applications.

## Figures and Tables

**Table 1:** The antioxidant activity and total polyphenol content (TPC) of medical-grade honeys (MGH) based on manuka honey (MH), chestnut honey (ChH), and multifloral honey (MFH). Artificial honey (AH) served as a negative control. Data represent the mean with standard deviation from three independent experiments. Different letters indicate significant differences between the tested MGHs; α = 0.05.

Method		MGH Type			AH
		MH	ChH	MFH	
DPPH	%	86.74 ± 1.13 a	84.67 ± 0.41 a	76.20 ± 1.13 b	3.95 ± 0.46 c
	mg TE/g	3.72 ± 0.08	3.63 ± 0.06	3.26 ± 0.01	0.12 ± 0.02
ABTS	%	77.84 ± 1.07 a	69.11 ± 0.65 b	63.99 ± 1.19 c	1.97 ± 1.36 d
	mg TE/g	16.45 ± 0.23	14.51 ± 0.14	13.37 ± 0.26	0.00 ± 0.00
PC (mg/L)		332.56 ± 1.74 a	358.37 ± 3.53 a	149.93 ± 6.38 b	nd

TE, Trolox Equivalent; nd, not detected.

**Table 2:** The composition of selected polyphenols of medical-grade honeys (MGHs) based on manuka honey (MH), chestnut honey (ChH), and multifloral honey (MFH). Data represent the mean with standard deviation from three independent experiments. Statistical analyses were performed using a one-way ANOVA using Tukey’s test. Different letters indicate significant differences in the content of individual polyphenols among the tested MGHs (α = 0.05).

Compound (µg/g)	MGH Type		
	MH	ChH	MFH
gallic acid	101.09 ± 5.82 a	54.21 ± 2.20 b	8.53 ± 0.31 c
ellagic acid	10.51 ± 1.41 a	5.76 ± 0.65 b	9.03 ± 0.63 a
ferulic acid	6.41 ± 0.13 a	3.91 ± 0.87 b	2.93 ± 0.25 b
chlorogenic acid	6.93 ± 1.64	nd	nd
vanillic acid	9.68 ± 0.44	nd	nd
protocatechuic acid	13.05 ± 0.91 b	26.66 ± 2.40 a	14.21 ± 2.28 b
gentistic acid	6.70 ± 1.54 a	7.23 ± 1.55 a	nd
caffeic acid	4.73 ± 1.39 a	3.30 ± 0.87 a	nd
*p*-hydroxybenzoic acid	7.82 ± 0.58	nd	nd
*m*-hydroxybenzoic acid	12.59 ± 0.52 a	13.11 ± 0.69 a	5.72 ± 0.26 b
rutin	11.77 ± 1.62 b	16.26 ± 0.95 a	5.91 ± 0.51 c
quercetin	nd	1.95 ± 0.40 a	2.17 ± 0.20 a

nd, no detected.

**Table 3:** Permeation of selected polyphenols from medical-grade honeys (MGHs) based on manuka honey (MH), chestnut honey (ChH), and multifloral honey (MFH) after 3, 5, 8, and 24 h of incubation. Data represent the mean with standard deviation from three independent experiments. Statistical analyses were performed using a one-way ANOVA using Tukey’s test.

Time (h)	Phenolic Compound (µg/cm^2^)											
	GA	EA	FA	ChA	VA	PrA	GeA	CA	PaA	mA	Rut	Qe
Manuka honey-based medical-grade honey (MH)												
3	3.07 ± 0.38	nd	nd	nd	nd	nd	0.44 ± 0.07	nd	nd	nd	nd	nd
5	4.26 ± 0.60	nd	1.73 ± 0.16	3.85 ± 0.81	nd	1.01 ± 0.09	0.65 ± 0.13	nd	nd	nd	nd	nd
8	5.32 ± 0.74	nd	2.57 ± 0.26	9.17 ± 2.74	nd	1.15 ± 0.13	0.94 ± 0.11	nd	nd	nd	nd	nd
24	17.58 ± 2.16	4.70 ± 0.17	3.58 ± 0.30	20.35 ± 0.85	0.33 ± 0.04	5.30 ± 1.00	4.64 ± 0.50	nd	48.17 ± 2.68	12.28 ± 0.33	5.30 ± 1.00	nd
Chestnut honey-based medical-grade honey (ChH)												
3	nd	nd	nd	nd	nd	nd	nd	nd	nd	nd	nd	nd
5	0.78 ± 0.15	nd	nd	nd	nd	nd	nd	nd	nd	nd	nd	nd
8	1.57 ± 0.11	4.91 ± 0.33	2.98 ± 0.44	nd	nd	nd	nd	nd	nd	nd	nd	nd
24	1.90 ± 0.15	11.07 ± 0.45	4.89 ± 0.68	nd	nd	1.45 ± 0.32	2.17 ± 0.60	nd	nd	11.51 ± 0.28	nd	nd
Multifloral honey-based medical-grade honey (MFH)												
3	nd	nd	nd	nd	nd	nd	nd	nd	nd	nd	nd	nd
5	nd	nd	nd	nd	nd	nd	nd	nd	nd	nd	nd	nd
8	nd	nd	nd	nd	nd	nd	nd	nd	nd	nd	nd	nd
24	nd	6.05 ± 0.31	2.03 ± 0.22	nd	nd	0.85 ± 0.02	nd	nd	nd	11.25 ± 0.39	nd	nd

GA, gallic acid; EA, ellagic acid; FA, ferulic acid; ChA, chlorogenic acid; VA, vanillic acid; PrA, protocatechic acid; GeA, gentistic acid; CA, caffeic acid; PaA, *p*-hydroxybenzoic acid; mA, *m*-hydroxybenzoic acid; Rut, rutin; Qe, quercetin; nd, not detected.

**Table 4:** Accumulation in the skin of selected polyphenols after application of medical-grade honeys (MGHs) based on manuka honey (MH), chestnut honey (ChH), and multifloral honey (MFH) after 24 h of incubation. The analysis was performed on skin extraction fluid collected after completion of the 24-h skin examination. Data represent the mean with standard deviation from three independent experiments. Statistical analyses were performed using a one-way ANOVA using Tukey’s test.

MGH	Content of Phenolic Compounds in the Skin (µg/g of skin)											
	GA	EA	FA	ChA	VA	PrA	GeA	CA	PaA	mA	Rut	Qe
MH	77.50 ± 6.40	17.09 ± 3.22	7.37± 2.01	31.39 ± 5.46	0.71 ± 0.20	20.46 ± 1.98	4.13 ± 2.31	2.73 ± 0.76	39.92 ± 5.10	24.82 ± 1.19	34.68 ± 2.42	nd
ChH	7.83 ± 0.68	17.29 ± 1.44	17.46 ± 2.61	nd	nd	13.01 ± 1.05	3.73 ± 1.21	nd	nd	53.28 ± 6.80	29.23 ± 1.94	nd
MFH	3.47 ± 0.30	12.40 ± 1.35	6.83 ± 1.13	nd	nd	5.22 ± 0.71	nd	nd	nd	31.43 ± 1.00	nd	nd

MGH, medical-grade honey; MH, manuka honey; ChH, chestnut honey; MFH, multifloral honey; GA, gallic acid; EA, ellagic acid; FA, ferulic acid; ChA, chlorogenic acid; VA, vanillic acid; PrA, protocatechic acid; GeA, gentistic acid; CA, caffeic acid; PaA, *p*-hydroxybenzoic acid; mA, *m*-hydroxybenzoic acid; Rut, rutin; Qe, quercetin; nd, not detected.

## Data Availability

Data is contained within the article or Supplementary Materials.

## Supplementary Materials

The following supporting information can be downloaded at: https://www.mdpi.com/article/10.3390/molecules31111863/s1, Figure S1: Honey cytotoxicity quantified by the MTT assay, Figure S2: HPLC chromatograms of medical-grade honeys (MGHs) based on (A) manuka honey (MH), (B) chestnut honey (ChH), and (C) multifloral honey (MFH), Table S1: Profile of nonpolar compounds in medical-grade honeys (MGHs) based on manuka honey (MH), chestnut honey (ChH), and multifloral honey (MFH) determined by GC-MS, Table S2: The composition of selected minerals, vitamin C, and sugar of medical-grade honeys (MGHs) based on manuka honey (MH), chestnut honey (ChH), and multifloral honey (MFH).
