# Supplementary material for: Botanical Origin-Dependent Phytochemical Profiles and Pharmaceutical Properties of Medical-Grade Honeys: Transdermal Delivery and Antibacterial Efficacy in a Wound Fluid Model
Source: Molecules. 2026 May 29;31(11):1863. doi: 10.3390/molecules31111863 (PMC13257883; doi:10.3390/molecules31111863)
Supplement: Supplementary file 1 [file molecules-31-01863-s001.zip › molecules-4308470-supplementary.pdf]

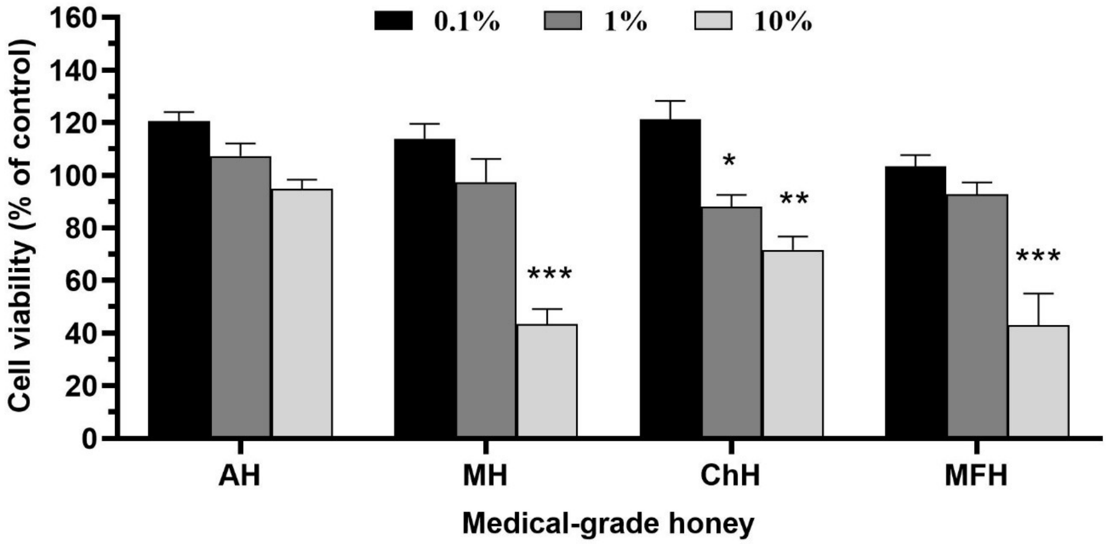

Figure S1. Honey cytotoxicity quantified by the MTT assay. Data represent a mean with standard deviation from three independent experiments. Statistical significance compared to the control was determined using ANOVA followed by Tukey's post-hoc test. *$P<0.05$.

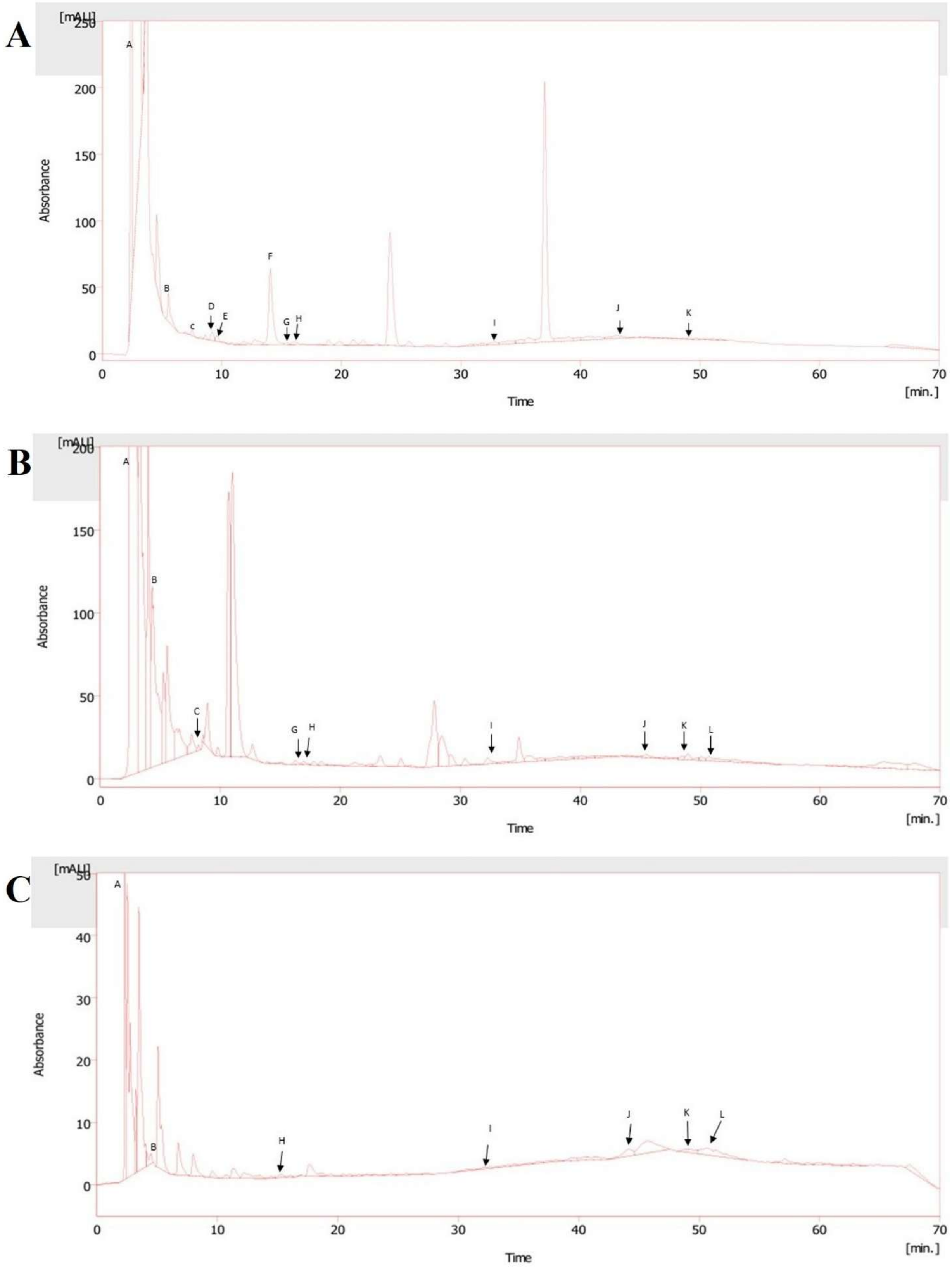
A
B
C
[mAU]
Absorbance
Time
[min.]
A
B
c
D
E
F
G
H
I
J
K
L

Figure S2. HPLC chromatograms of medical-grade honeys (MGHs) based on (A) manuka honey (MH), (B) chestnut honey (ChH), and (C) multifloral honey (MFH); A- gallic acid, B- protocatechuic acid, C- gentistic acid, D- p-hydroxybenzoic acid, E – chlorogenic acid, F – vanilic acid, G- caffeic acid, H - m-hydroxybenzoic acid, I- ferulic acid, J – elagic acid, K – rutin, L - quercetin

Table S1. Profile of nonpolar compounds in medical-grade honeys (MGHs) based on manuka honey (MH), chestnut honey (ChH), and multifloral honey (MFH) determined by GC-MS.

Compound	MH	ChH	MFH	Summary formula	CAS
Cyclopentane, 1,2,3-trimethyl- (Beeswax, lipids)	x			C_8H_{16}	3074-71-3
Cyclohexane, methyl- (Waxes, lipids)	x	x	x	C_7H_{14}	108-87-2
Dodecane, 4,6-dimethyl- (Beeswax, lipids)	x			$C_{14}H_{30}$	17301-94-9
Tetradecane (Beeswax, lipids)	x		x	$C_{14}H_{30}$	629-59-4
3-Ethyl-2,6,10-trimethylundecane (Isoprenoids, plant origin)	x	x	x	$C_{16}H_{34}$	3891-98-3
Heneicosan (Beeswax, lipids)	x		x	$C_{21}H_{44}$	629-94-7
Eicosane (Beeswax, lipids)	x	x	x	$C_{20}H_{42}$	112-95-8
Tridecanoic acid, 4,8,12-trimethyl-, methyl ester (Fatty acid esters)	x			$C_{17}H_{34}O_2$	5129-60-2
Dotriacontane (Beeswax)	x		x	$C_{32}H_{66}$	544-85-4
Methyl stearate (Fatty acid esters)	x			$C_{19}H_{38}O_2$	112-61-8
Tetrapentacontane (Beeswax)	x			$C_{54}H_{110}$	13550-08-8
9-Octadecenamide, (Z)- (Oleic acid amide, lipids)	x		x	$C_{18}H_{35}NO$	301-02-0
Tetracosan (Beeswax, lipids)	x	x		$C_{24}H_{50}$	646-31-1
Heptadecane, 3-methyl- (Waxes, lipids)	x	x	x	$C_{18}H_{38}$	6418-44-6
Palmitoleamide (Fatty acid amide, lipids)	x	x		$C_{16}H_{31}NO$	112-84-5
Carbonic acid, decyl pentadecyl ester (Fatty acid esters)		x		$C_{26}H_{52}O_3$	-

3-Ethyl-3-methylheptane (Izoprenoidy, lipidy)	x	$C_{10}H_{22}$	62016-18-0
Hexadecane, 2,6,10,14-tetramethyl- (Beeswax, lipids)	x	$C_{20}H_{42}$	1921-70-6
1,3-Propanediol, ethyl tetracosyl ether (Fatty acid esters, lipids)	x	$C_{29}H_{60}O_3$	-

Table S2. The composition of selected minerals, vitamin C, and sugar of medical-grade honeys (MGHs) based on manuka honey (MH), chestnut honey (ChH), and multifloral honey (MFH). Data represent the mean with standard deviation from three independent experiments. Statistical analyses were performed using a one-way ANOVA using Tukey's test. Different letters indicate significant differences in the content of individual polyphenols among the tested MGHs (α = 0.05).

**Compound/Element** (µg/g)	**MGH type**		
	**MH**	**ChH**	**MFH**
ascorbic acid	63.00 ± 1.00b	75.67 ± 1.53a	26.00 ± 0.00c
iron	< 0.5	< 0.5	< 0.5
magnesium	9.00 ± 0.00b	20.00 ± 0.00a	7.00 ± 6.33c
calcium	13.00 ± 0.00c	32.00 ± 0.00a	20.00 ± 0.00b
chloride	< 0.5	< 0.5	< 0.5
Lactic acid	16.53 ± 0.74b	17.37 ± 0.12b	24.10 ± 0.31a
sugar (G+C)	140700.00 ± 519.62d	178100.00 ± 458.26c	185900.00 ± 3411.74b
sucrose	< 0.25c	< 0.25c	0.71 ± 0.02b
